# Metformin: A Bridge between Diabetes and Prostate Cancer

**DOI:** 10.3389/fonc.2017.00243

**Published:** 2017-10-11

**Authors:** Veronica Zingales, Alfio Distefano, Marco Raffaele, Antonio Zanghi, Ignazio Barbagallo, Luca Vanella

**Affiliations:** ^1^Department of Drug Science, Biochemistry Section, University of Catania, Catania, Italy; ^2^Department of Surgery, Azienda Ospedaliero Universitaria Policlinico Vittorio Emanuele, Catania, Italy

**Keywords:** diabetes, metformin, prostate, cancer, apoptosis

## Abstract

Prostate cancer (PCa) has become the most frequent type of cancer in men. Recent data suggest that diabetic patients taking metformin have a lower incidence of certain cancer, including PCa. Metformin is the most common drug used in type II diabetes mellitus; its use has been shown to lower the incidence of several cancers, although there are ambiguous data about the anticancer activity of metformin. A large number of studies examined the potential antineoplastic mechanism of metformin although it is not still completely understood. This review summarizes the literature concerning the effects of metformin on PCa cells, highlighting its numerous mechanisms of action through which it can act. We analyze the possible causes of the discordances regarding the impact of metformin on risk of PCa; we discuss the latest findings in this field, suggesting that metformin may have a future role in the management of PCa both as monotherapy and in combination with other drugs.

## Introduction

Prostate cancer (PCa) represents one-third of all new cancer cases each year and the second cause of cancer-related death in US (1).

Prostate cancer is the stage subsequent to premalignant lesions due to a progressive transition from normal prostatic epithelial cells to prostatic intraepithelial neoplasia. Over time, most tumors evolve in castration-resistant prostate cancer (CRPC) with development of metastasis (2, 3). Advanced stages of the disease and formation of metastasis are the main causes of most PCa-related death. Multiple treatment strategies exist but the survival rates remain low. The current strategies for the management of PCa include surgery, chemotherapy, radiation, and endocrine therapy.

In the early stage, PCa is characterized by androgen-dependent growth and medical castration through androgen-deprivation therapy (ADT) is the first-line therapy choice for its treatment. This therapy depresses the proliferative function of androgen receptor (AR) but after 12 and 18 months, patients treated with ADT develop resistance to this therapy (4). In fact, over time, androgen-dependent PCa evolves to androgen-independent PCa and ADT is not useful to treat the progressive stage of PCa. CRPC is the most aggressive form of PCa and it shows resistance to current available therapeutic strategies. Recent studies demonstrated the existence of a relationship between diabetes, insulin levels, and risk of cancer, including PCa, but other studies, investigating the association between diabetes mellitus and PCa, have reported inconsistent findings (5). Notably, although the PCa affects a considerable proportion of men, a reduced incidence of this type of cancer has been observed in subject with type II diabetes mellitus (T2DM). Probably, lower hormone levels, such as testosterone, insulin, and insulin-like growth factor-1 (IGF-1) in patients with uncontrolled T2DM induces a lower risk of developing PCa (6). Although pieces of epidemiological evidence showed that diabetes mellitus is inversely associated with the development of PCa (6, 7), other studies proved opposite results (8–10). Circulating insulin levels, higher than average, have been shown to be associated with increased cancer growth and mortality (11–13). This could be explained by analyzing the role of insulin and/or IGF-1 on insulin-like growth factor-1 receptor (IGF-1R). High levels of IGF-1R are associated with invasion, aggressiveness cancer, and poorer prognosis (14–18).

In addition to the escalation of the incidence of PCa, over the last three decades, an ever-going epidemic of diabetes occurred particularly in developed countries. World Health Organization estimated that diabetes will become one of the main causes of death in the world by 2030 (19).

Metformin is an insulin-sensitizing oral biguanide used by diabetic patients every day to maintain their glycemic homeostasis. Metformin is an ideal drug: it is well tolerated and inexpensive. Metformin regulates glucose homeostasis exerting an important control of metabolism. In particular, metformin reduces intestinal absorption of glucose and it increases peripheral glucose uptake and its utilization by adipose tissue and skeletal muscles leading to increased insulin sensitivity. Through AMPK activation, metformin decreases insulin secretion, inhibits gluconeogenesis and energy consuming processes (such as protein and fatty acid synthesis), and stimulates ATP-generating processes (such as glycolysis and fatty acid oxidation). This results in a shift from anabolic to catabolic metabolism and in an inhibition of proliferation. Metformin and a safety lifestyle, the latter based on healthy and well-balanced meals and a regular physical activity, are currently the first-line treatment for the management of T2DM.

## The Antineoplastic Effects of Metformin

Recent studies were interested to investigate whether metformin might have potential benefits on other widespread diseases, such as the cancer, suggesting a new potential use of this drug beyond its classical indications. In fact, it seems that metformin owns antineoplastic activity and this ability might be the cause of the inverse relationship between diabetes and risk of developing PCa. This hypothesis remains credible and should continue to be investigated. Recently, metformin has been shown to exert antineoplastic effects in several cancers. Its anticancer activity has been highlighted by diabetes specialists who noticed diabetic patients taking metformin had a lower risk to develop cancer (including PCa) than other diabetic patients. The role of metformin in glucose and fatty acids metabolism is very well known but further studies are needed to explore the cellular mechanism and its cell targets in cancer cells.

In the last years, many researchers focused the attention on the metformin and several studies and meta-analysis were conducted. These studies showed an unclear role of metformin on prevention of PCa: some studies showed that use of metformin is associated with a reduced risk of development of PCa; other studies showed that this association does not exist (20, 21).

A retrospective cohort study showed that metformin use is associated with a reduced risk of PCa in Asian patients with type II diabetes (22). Meta-analysis by Hwang et al. revealed that metformin use causes a 20% reduction in risk of recurrence in PCa patients with T2DM (23).

Another large retrospective cohort study did not find an association between metformin use and risk of PCa in men with diabetes, regardless of cancer grade (high-grade and low-grade cancer) or diagnosis method (24).

The effect of metformin on the prognosis of patients with cancer was also investigated, showing a statistically significant decrease in cancer-related mortality, following metformin use (25). In addition, in patients with PCa, metformin use was associated with a lower risk of death due to PCa, and the benefit increased in a way that is dependent on the duration of its use (26).

Other studies showed different results (27) and these discrepancies could be explained by differences in patient characteristics, different origin, grade, and biological features of PCa and dose and duration of drug treatment (metformin seems to require long-term use to exert its anticancer activity) (28, 29). However, from a large number of studies, metformin results to have anticancer activity, decreasing the incidence of different types of cancer, such as colon, breast, pancreatic, and PCa *in vivo* and *in vitro* (30–34).

## AMPK-Dependent and Independent Molecular Mechanism

AMPK activation appears the main mechanism through which metformin inhibits cancer growth. AMPK plays a key role in the maintaining of cellular energy homeostasis (35). It is an important sensor of the AMP/ATP ratio. AMPK appears as a potential anticancer agent when it is highly activated, but it may not be critical as inhibitor of cancer growth when it acts at low levels (36).

Metformin primarily acts to directly inhibit the mitochondrial respiratory chain which then reduces the production of ATP resulting in an increase in the ratio of AMP to ATP which then results in activation of AMPK. Under energy stress conditions, the tumor suppressor LKB1 (37) is the major kinase involved in the AMPK activation and mTOR reduction.

Through the mTOR inhibition, metformin arrests cell cycle and cell growth, because mTOR is a downstream effector of PI3K/AKT pathway, a signaling pathway linked to cancer cell growth and proliferation. PI3K/AKT/mTOR signaling pathway leads to an abnormal cells proliferation, inhibition of apoptosis, and carcinogenesis.

Colquhoun et al. showed that metformin owns an anti-proliferative effect in PCa cells through the activation of pAMPK and subsequent inhibition of downstream mTOR signaling and the induction of cell cycle arrest. In this study, metformin was used in combination with bicalutamide, a known agent used in the hormonal therapy of PCa. It acts blocking the AR and inducing a G1/S phase arrest of the cell cycle. Combining metformin with bicalutamide, the authors obtained a reduction of PCa cell survival, especially in cells expressing functional AR (38).

The anti-PCa effect of metformin *via* AMPK activation was also observed by Tsutsumi et al. They demonstrated, *in vitro* and *in vivo*, that metformin induces apoptosis and attenuates PCa cell proliferation. Furthermore, a stronger decrease of PCa growth was achieved when metformin was combined with Exenedin-4, a glucagon-like peptide-1 receptor agonists (39).

A key feature of cancer cells is an increase in dependency on glycolysis for energy production. This is known as “Warburg effect.” 2-Deoxyglucose (2DG) has been considered a potential anticancer agent for its capacity to inhibit glucose metabolism and induce intracellular ATP reduction and autophagy (40). In the study conducted by Ben Sahra et al., the combination of metformin and 2DG resulted in a synergistically effect on cell viability in PCa cells, due to a stronger depletion of intracellular ATP, through the attack to two different sources of energy: mitochondrial complex 1 and glycolysis (41).

Although AMPK activation appears the main mechanism of action through which metformin exerts its anticancer effect, other antineoplastic mechanisms of action AMPK-independent have been shown.

In DU145 LKB1-negative cells, Biernacka et al. did not observe an increased phosphorylation of AMPK after treatment with metformin, suggesting that, in this specific cell line, metformin induces cell death through a LKB1–AMPK-independent pathway (42).

In addition, Ben Sahra et al. reported that REDD1 and cyclin D1, in an AMPK-independent manner, mediate the anti-proliferative effects of metformin in PCa cells (43, 44).

In addition, it was demonstrated that metformin treatment decreases c-MYC protein levels and the incidence of prostate intraepithelial lesions formation *in vivo* and *in vitro* (45). The levels of c-MYC protein and mRNA in the metformin-treated PCa cells were much lower than those in control cells. Furthermore, in LNCaP cells, androgen-sensitive human PCa cells are characterized by the presence of high-affinity AR (46); the reduction of c-MYC levels was associated with a significant reduction of AR.

Activated AR binds androgen response element and regulates the expression of genes involved in PCa cell growth.

Metformin is able to downregulate the levels of AR mRNAs, showing another way to repress the cancer cell proliferation (47). Furthermore, through its ability to downregulate AR, metformin inhibited the migration both AR-negative and AR-positive PCa cells, showing more pronounced effect in these last. The authors suggest that the mechanism through which metformin abrogates the upregulation of AR is *via* enhanced activity of the MID1 translation regulator complex.

In PCa cells, androgens induce a selective upregulation of IGF-1R, with a subsequent increase of cell proliferation and invasiveness (48). Standard anti-androgen drugs are not capable to block the IGF-1R upregulation. Malaguarnera et al. showed that metformin is able to inhibit androgen-dependent IGF-1R upregulation with subsequent reduction of IGF-1R-mediated proliferation in LNCaP cells (49). This study found that the complex more involved in the androgen-dependent IGF-1R upregulation is the mTORC1 complex, whereas AMPK plays a marginal role in this action.

Since androgens increase proliferation and development of PCa cells, ADT is the first line of PCa treatment. However, after several years, many patients do not well response to this therapy, developing castration-resistant prostate cancer (CRPC). Furthermore, ADT can cause metabolic consequences, such as insulin resistance and development of metabolic syndrome (50). A combination of metformin with androgen deprivation might improve treatment efficacy and minimize side effects.

Some studies showed that ADT might provide microenvironments suitable for the differentiation of cancer cells in hormone-independent cancer cells, increasing factors involved in the epithelial–mesenchymal transition (EMT) and exerting a selective pressure toward EMT (51, 52). Understanding the molecular mechanism by which EMT acts in cancer progression, identifying agents capable of stopping or slowing metastasis, may be an essential step for PCa treatment. Some studies highlighted that COX2/PGE2/STAT2/EMT is involved in cancer cell migration and invasion (53–56). Tong et al. showed that metformin is capable to act on this axis, inhibiting it. In fact, their data revealed that metformin has an anti-EMT effect through the inhibition of COX2 or the block of PEG2-mediated STAT3 phosphorylation and the expression of other EMT markers, when metformin is used at higher concentrations (57). They also demonstrated that metformin is capable, by inhibiting EMT, to restore enzalutamide sensitivity in CRPC (58).

Another known factor associated with EMT in lung fibrosis and breast cancer is FoxM1 (59, 60), but its role in PCa cells has not yet been elucidated. FoxM1 plays a key role in cell proliferation, cell cycle regulation, angiogenesis, invasion, and metastasis (61, 62). Wang et al. found that in PCa cells EMT is inhibited by metformin through the downregulation of FoxM1 expression (63).

Metformin has been shown to inhibit EMT by modulating microRNA, such as miR30a (64). In recent years, the involvement of miRNAs in human PCa has been investigated, discovering several miRNAs expressed abnormally (65, 66). Among these, miR-708-5p is a non-coding RNA with a tumor- and metastasis-suppressive role (67, 68). Yang et al. showed that metformin upregulates miR-708-5p in LNCaP and PC3 cells, inducing ER stress and apoptosis (69). During the early and late stages, PCa is characterized by an increase of lipogenesis that it is associated with tumor growth and the most aggressive forms of PCa (70). Metformin is able to alter the activity and the expression of lipogenic enzymes and transcriptional factors, such as SREBP-1c, FAS, and ACC, causing an energy deficiency (71). The capacity of metformin to decrease the expression of FAS and SREBP-1c is linked with its ability to act on AMPK/mTOR pathway.

Therefore, it has been shown that metformin acts on multifold molecular targets and on a myriad of transduction pathway.

In recent experimental studies, metformin is often used at concentrations clearly higher than those observed in diabetic patients, but it has been demonstrated that many organs and tissues are exposed to metformin concentrations appreciably higher than those present in the general circulation (72). Furthermore, metformin has been shown to have irrelevant effects on benignant cells, suggesting that its pro-apoptotic effects are limited to malignant cells (45).

## The Combination of Metformin with Other Drugs or Natural Agents

Researchers evaluated the effect of metformin in combination with other drugs or natural agents on PCa cells. Metformin exerts its antineoplastic effect at lower concentrations when it is used in combination with other agents, such as Plk1 inhibitor. Shao et al. suggest that the inhibition of Plk1 promotes the cytotoxicity of metformin in PCa cells through both signaling and metabolic pathways (73).

In addition to metformin, statins are also the most commonly prescribed drugs in western countries. Recent data suggest that statins may have beneficial effects against PCa (74). The role of the combination of metformin and simvastatin was studied by Pennanen et al., showing a synergistically increase of necrotic cell death and autophagy (75).

Moreover, in the last years, natural-derived and medical plants have acquired more and more importance, such as solamargine (76, 77). The capacity of solamargine alone and in combination with metformin on CRPC cells has been explored (78). This study showed that solamargine inhibits the growth of two CRPC cell lines (DU145 and PC3) in the dose-dependent manner; this antineoplastic effect of solamargine is enhanced by metformin. The mechanism through which the combination of two agents reduces the proliferation of CRPC cells consists in the AMPKα-mediated inhibition of p65. A recent study showed that the AMPK-dependent antineoplastic effect of metformin results stronger when it is used in combination with Vitamin D3 (79). This study suggests that vitamin D3 improves metformin’s inhibitory activity due to AMPK activation and G1/S cell cycle arrest. In addition, metformin and vitamin D3 treatment has been shown to reduce anti-apoptotic protein Bcl-2 levels (with subsequent increase of cellular apoptosis) and c-MYC expression in DU145 more than the single treatment, highlighting a synergistic effect of the combination of the two agents.

## Conclusion

**Figure 1:**
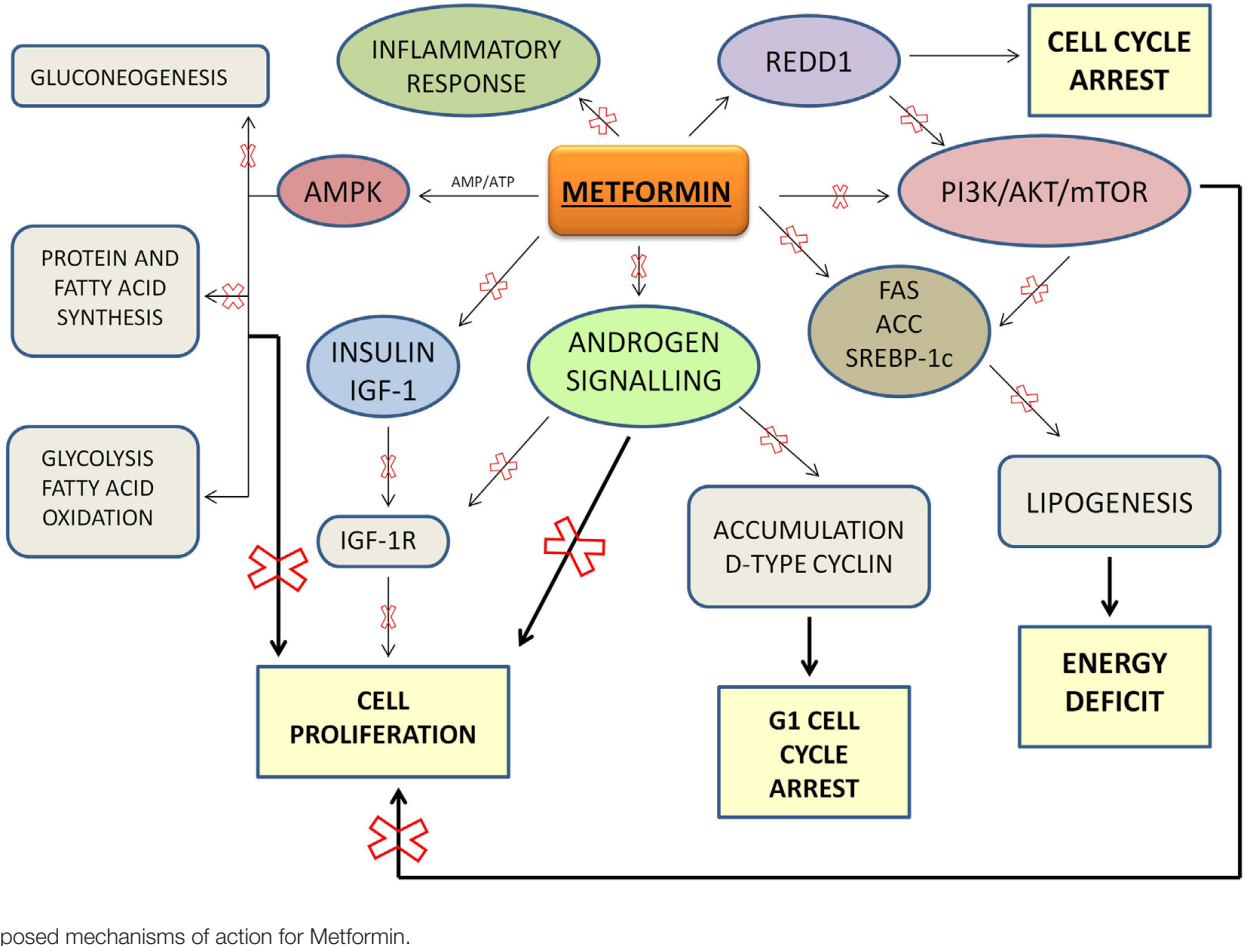
Proposed mechanisms of action for Metformin.

Overall, metformin may be considered an ideal agent to be used as adjuvant to standard treatments for PCa, both as monotherapy and combined with chemotherapeutics or other drugs (Table 1). Current treatments for PCa result limited because of drug resistance and toxicity that develop over time. Thus, new cellular targets and novel molecular therapeutic agents are needed. To this regard, metformin appears a real candidate more affordable than other expensive therapeutic options: metformin plays a central role as metabolic homeostasis regulator and indirectly as anti-proliferative and anti-carcinogenic agent (Figure 1). Sufficient data from studies on cancer cell lines and animal models suggest that metformin lowers the risk of biochemical recurrence and the rates of mortality in PCa, through its intrinsic proprieties and its pleiotropic effects linked with metformin-mediated fall in plasma glucose and insulin concentrations.

**Table 1:** Characteristics of selected study.

Metformin and prostate cancer (PCa)				
Compound	Type of study	Cell culture system or animal model	Concentration used	Mechanism of action
Metformin + bicalutamide	*In vitro* and *in vivo* (38)	DU145, PC3, LNCaP LNCaP xenograft murin model	10 mM	Activation of pAMPK
Metformin + exendin-4	*In vitro* and *in vivo* (39)	DU145, PC3, LNCaP Athymic CAnN. Cg-*Foxn1nu*/CrlCrlj non-diabetic male mice	Metformin: 0.1–10 mM Ex-4: 10 nM	Activation of pAMPK
Metformin + 2-deoxyglucose (2DG)	*In vitro* (41)	LNCaP, P69, PC3, DU145	Metformin: 1 mM, 5 mM 2DG: 1 mM	Activation of pAMPK
Metformin	*In vitro* (42)	LNCaP, PC3, DU145, VCaP	1–10 mM	Downregulation of IGFBP-2
Metformin	*In vitro* (44)	LNCaP, DU145, PC3	1–10 mM	Increase of REDD1 expression
Metformin	*In vitro* and *in vivo* (43)	DU145, PC3, LNCaP, P69 Mice bearing xenograft LNCaP	1 mM, 5 mM	Decrease of cyclin D1 level
Metformin	*In vitro* and *in vivo* (45)	Hi-Myc mouse Myc-CaP, C4-2b, PC3, LNCaP	200 mg/kg/day, 2 mM	Decrease of c-MYC protein level
Metformin	*In vitro* (47)	LNCaP, PC3, DU145, VCaP, RWPE-1	0.01–5 mM	Increase of activity of MID1 translational regulator complex and androgen receptor downregulation
Metformin	*In vitro* (49)	LNCaP, HEK293	3–30 mM	Inhibition of androgen-dependent insulin-like growth factor-1 receptor upregulation
Metformin	In humans, *in vivo* and *in vitro* (57)	32 samples from patients with PCa PC3, 22RV1	1–20 mM	Repression of epithelial–mesenchymal transition (EMT) by targeting the COX2/PGE2/STAT3 axis
Metformin + enzalutamide	*In vitro* and *in vivo* (58)	C4-2, LNCaP, CWR22Rv1 Castrated male nude mice	Metformin: 5 mM; 300 mg/kg/day Enzalutamide: 20 µM; 25 mg/kg/day	Inhibition of enzalutamide-induced EMT *via* TGF-β1/STAT3 axis
Metformin	*In vitro* (63)	LNCaP, DU145, PC3, PC3M	5–25 mM	Inhibition of EMT through the downregulation of FoxM1 expression
Metformin	*In vitro* (69)	LNCaP, PC3, C4-2B	5 mM	Upregulation of miR-708-5p
Metformin	*In vitro* (71)	LNCaP, PC3, DU145	5 mM	Inhibition of lipogenesis
Metformin + BI2536	*In vitro* and *in vivo* (73)	LNCaP, C4-2, DU145, PC3, HEK293A, RWPE-1 Nu/Nu nude mouse	Metformin: 0.5–5 mM; 5–15 mg/kg BI2536: 1–30 nM; 5–30 mg/kg	p53/Redd-1 pathway
Metformin + simvastatin	*In vitro* (75)	RWPE-1, LNCaP	Metformin: 0.1–1 mM Simvastatin: 100 nM, 1–5 µM	Induction of cell cycle block, autophagy and necrosis
Metformin + solamargine	*In vitro* and *in vivo* (78)	DU145, PC3, C4-2B, BPH-1 30 female nude mice	Metformin: 5 mM Solamargine: 1–10 µM; 5–10 mg/kg	AMPKα-mediated inhibition of p65
Metformin + vitamin D3	*In vitro* (79)	DU145	Mformin: 1,000–10,000 µg/ml Vitamin D3: 400–800 µg/ml	AMPK activation

Although the potential mechanism of action of metformin has been largely studied, it is not still completely understood. Other studies are necessary to determine which dose of metformin can cause profound direct antineoplastic effects on cancer cell metabolism and if these doses can be safely administered to patients.

## Author Contributions

VZ, AD, and MR collected and analyzed research article; AZ gave conceptual advise; and IB and LV wrote the manuscript.

## Conflict of Interest Statement

The authors declare that the research was conducted in the absence of any commercial or financial relationships that could be construed as a potential conflict of interest.
